# Supplementary material for: A non-classical view on calcium oxalate precipitation and the role of citrate
Source: Nat Commun. 2017 Oct 3;8:768. doi: 10.1038/s41467-017-00756-5 (PMC5626694; doi:10.1038/s41467-017-00756-5)
Supplement: Supplementary file 1 — Supplementary Information [file 41467_2017_756_MOESM1_ESM.pdf]

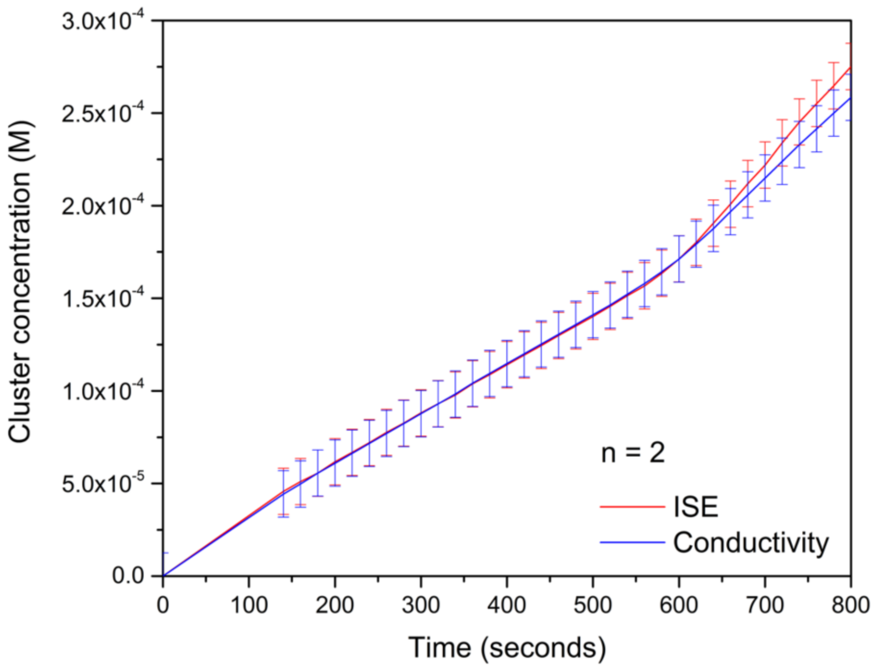

Supplementary Figure 1. Calculated Ca-oxalate cluster concentration. Red line show the calcium oxalate cluster concentration calculated from ISE measurements and the blue line that calculated using conductivity measurements and assuming a oxalate to calcium ratio of 2 (n) in the clusters. Error bars s.d.

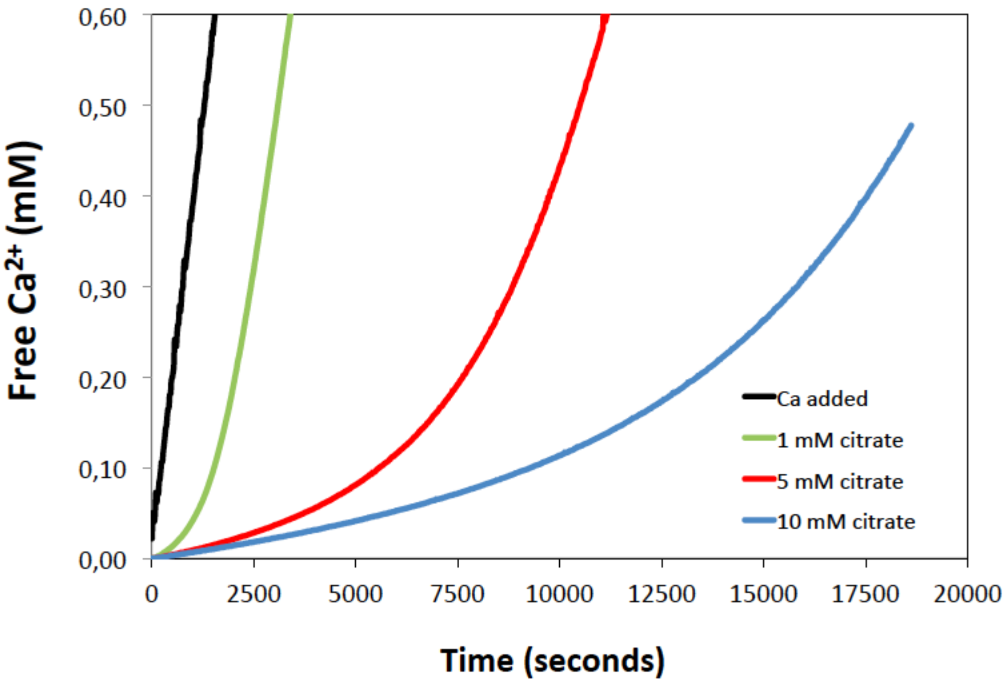

Supplementary Figure 2. Calcium complexation by citrate. Time development of the free Ca^{2+} concentration in titration experiments in aqueous solutions (no oxalate in the reaction media) in the presence of different concentrations of citrate at pH 6.2. The black line refers to the amount of Ca^{2+} added.

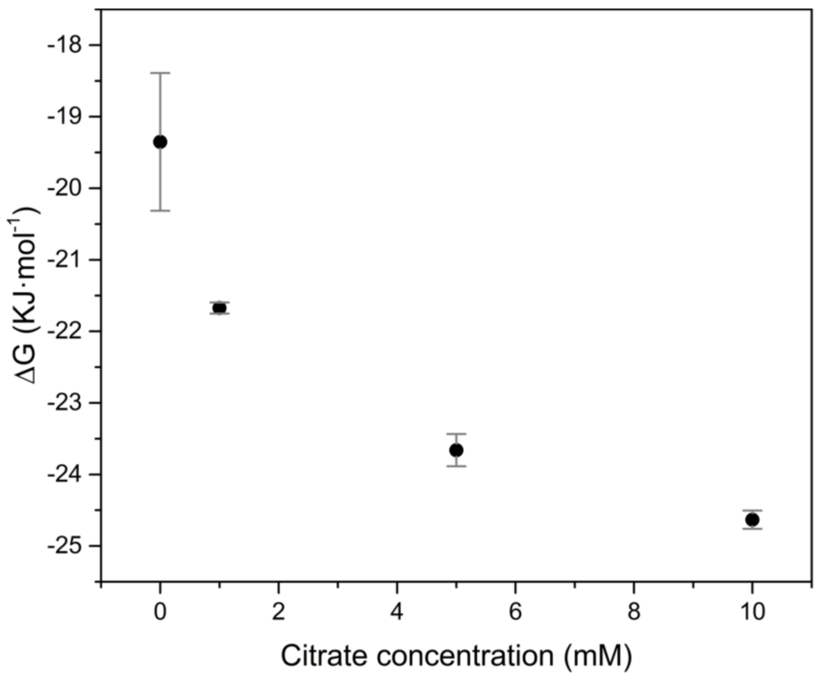

Supplementary Figure 3. Stability of calcium oxalate clusters as a function of citrate concentration. Gibbs standard energy for the formation of calcium/oxalate ion pairs in calcium oxalate clusters, as a function of citrate concentration.

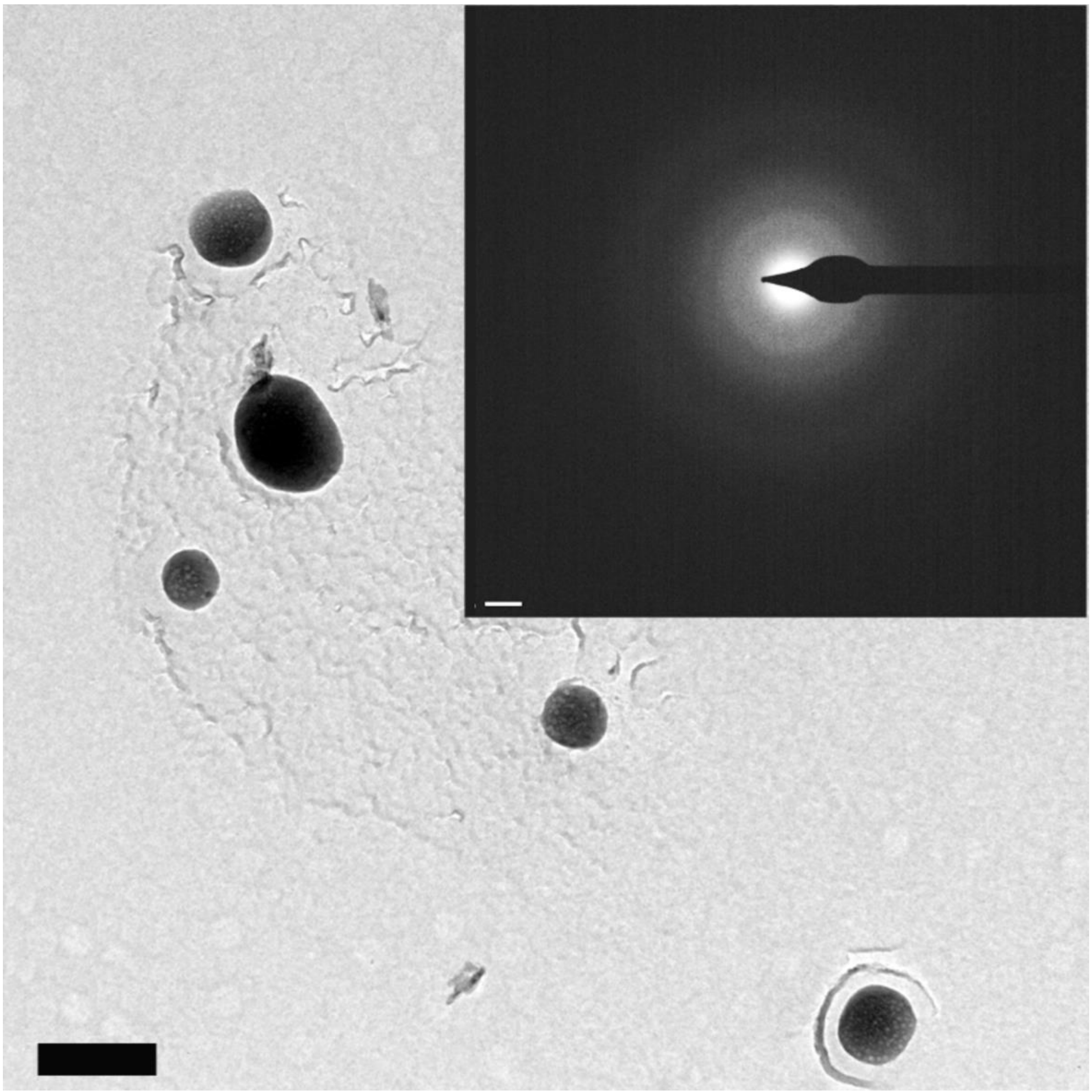

Supplementary Figure 4. Amorphous calcium oxalate in control runs. TEM photomicrograph of calcium oxalate nanoparticles formed after 1600 s in the titration experiments (control runs). SAED pattern showing their amorphous nature in inset. Scale bar: 200 nm. Scale bar in the inset: 2 nm^{-1}

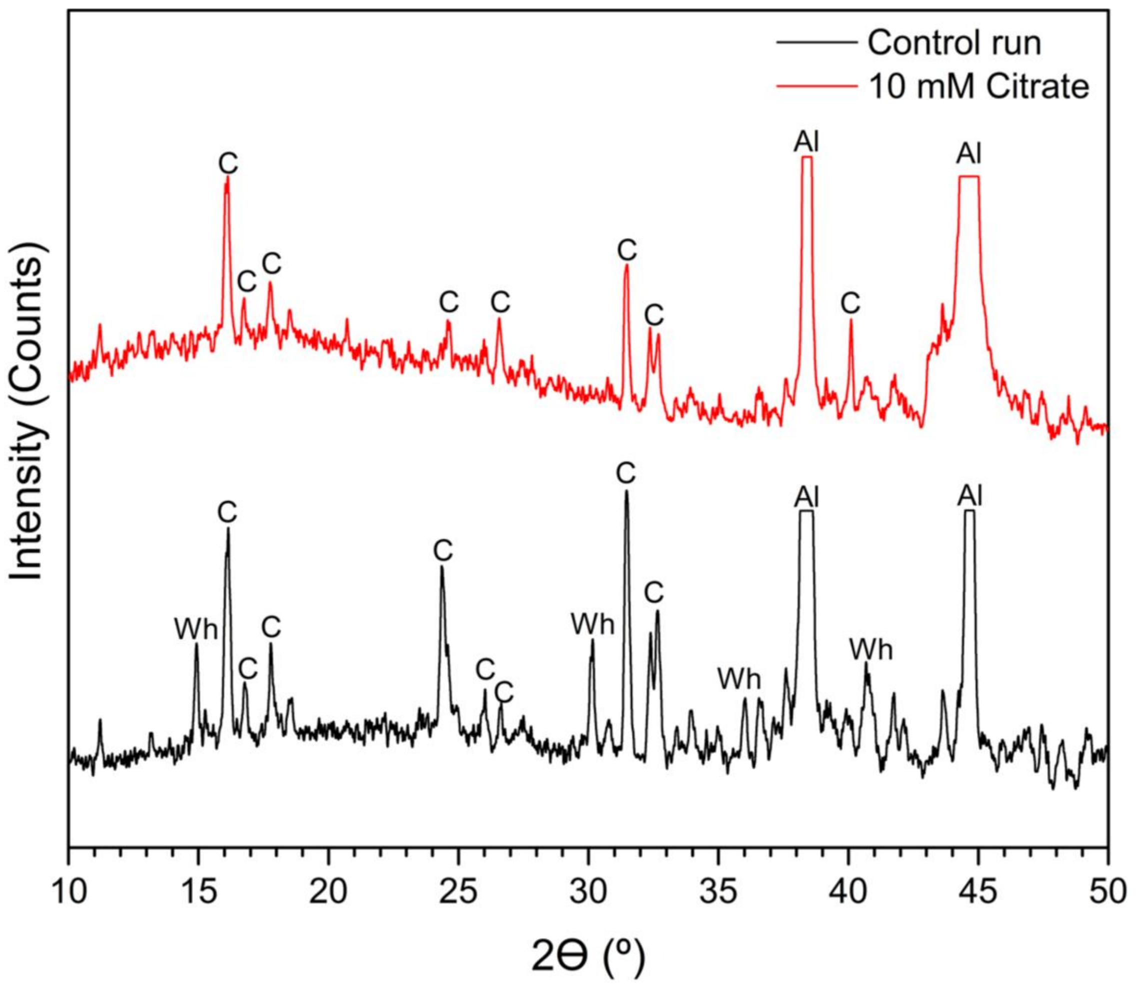

Supplementary Figure 5. X-Ray diffraction patterns of solids collected at the end of titration experiments. Black line: control run (i.e. no citrate in the reaction media). Red line: experiment performed in the presence of 10 mM citrate.

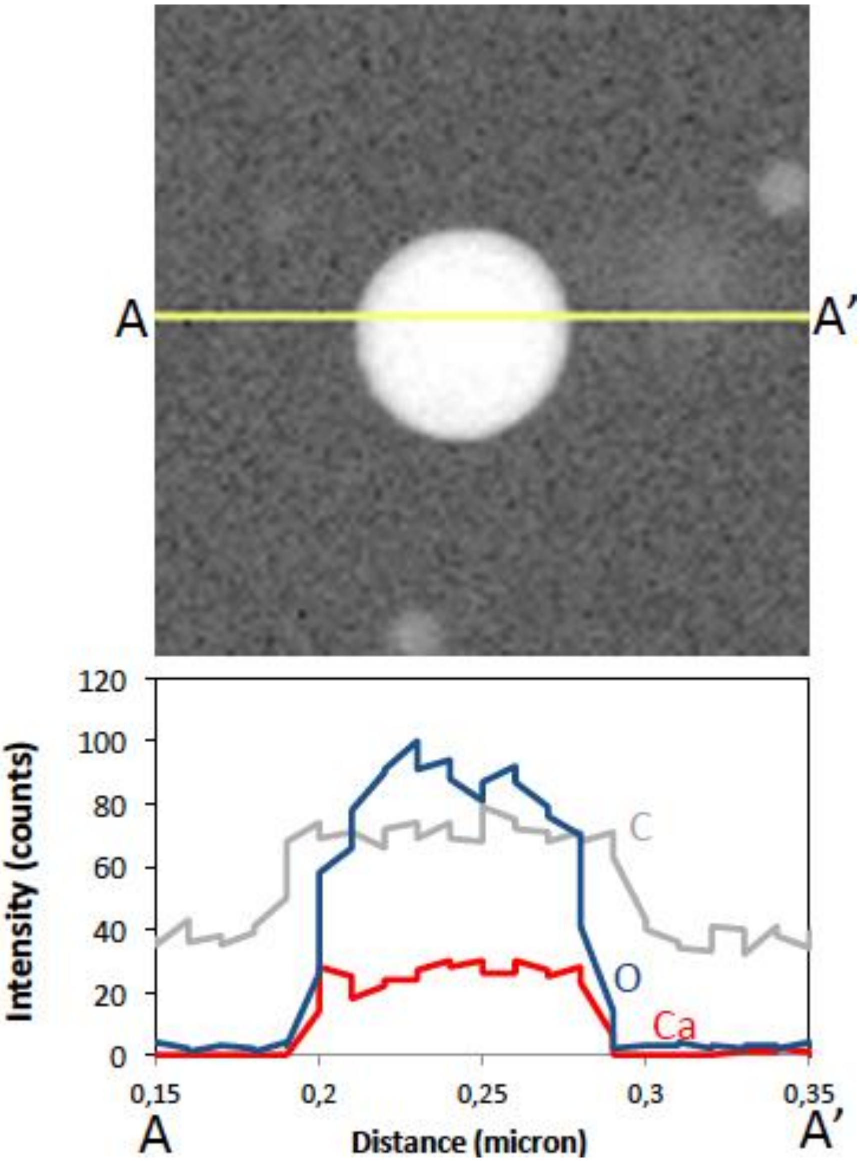

Supplementary Figure 6. STEM line profile analysis of ACO nanoparticles. TEM annular dark field images of the edge of an ACO nanoparticle displaying a ca. 20 nm thick rim and line-profile EDS analysis (along the line A-A’ depicted in the TEM image) showing an increase in C in the outer part of the sphere is likely due to the presence of an adsorbed/incorporated layer of citrate.

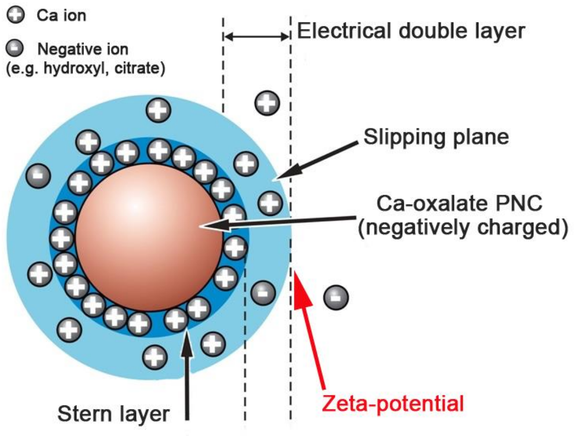

Supplementary Figure 7. Schematic diagram showing the ionic concentration around the negatively charged calcium oxalate clusters. Note that this is just a sketch to illustrate our model explaining the slightly positive zeta potential measure in the clusters in the absence of citrate, as well as citrate binding, as PNCs actually do not have surfaces that separate them from the surrounding solution.

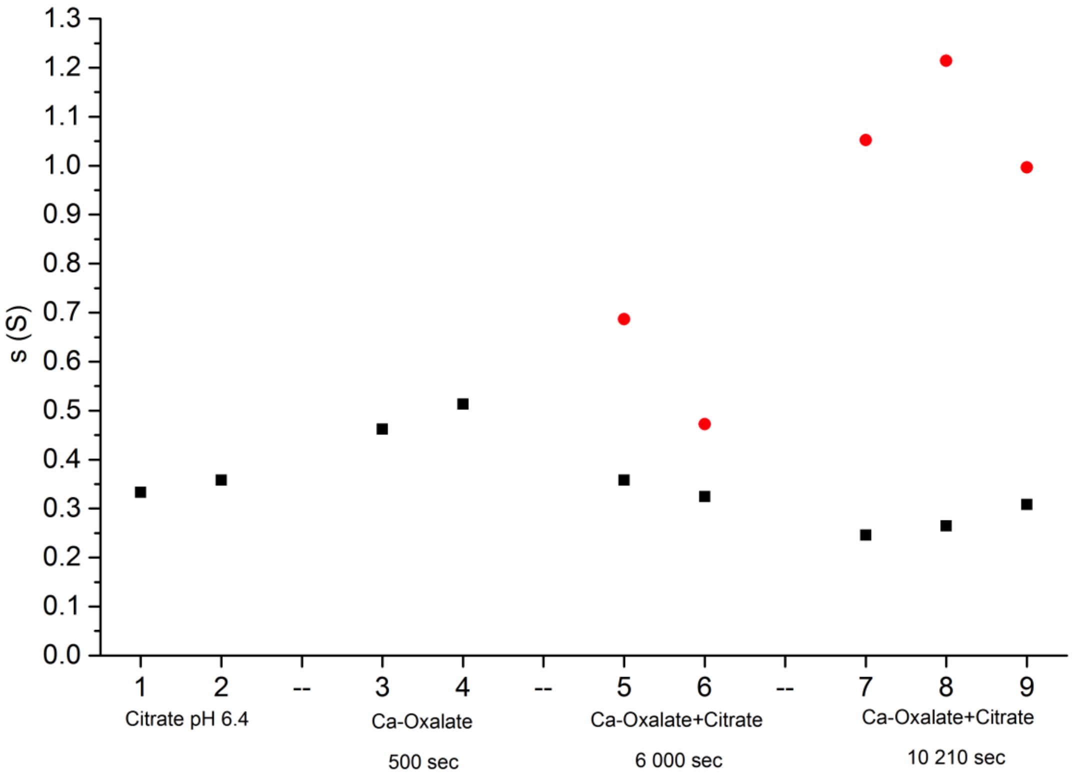

Supplementary Figure 8. Analytical Ultracentrifugation analysis (I). Sedimentation coefficients measured for samples drawn from citrate buffer pH 6.4 (1,2), control runs (no citrate in the reaction media) with samples taken after 500 s (3,4), titration runs performed in the presence of 10 mM citrate taken after 6000 s (5, 6) and 10210 s (7 - 9). Two different species were found: the black dots are the smallest species and the red ones are the next bigger species that are found in the presence of citrate.

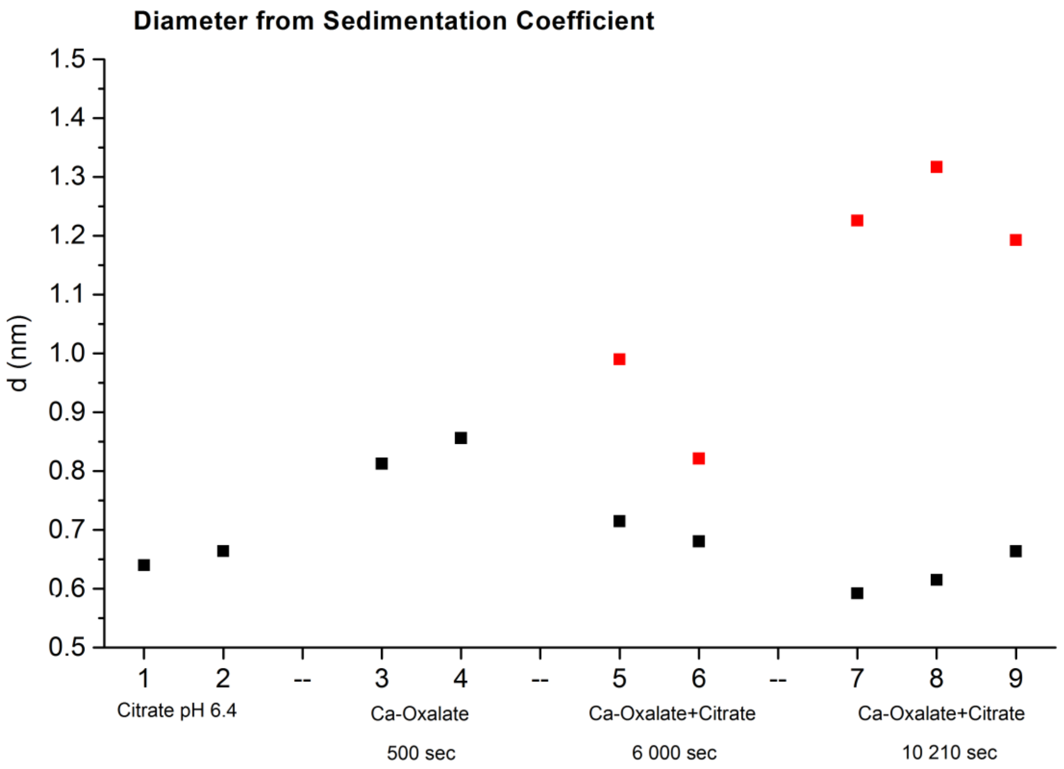

Supplementary Figure 9. Analytical Ultracentrifugation analysis (II). Apparent hydrodynamic diameters calculated from the sedimentation coefficients in figure S5 using $\overline{v}$ = 0.435 ml/g for citrate and $\overline{v}$ = 0.472 ml/g for CaC_2O_4.

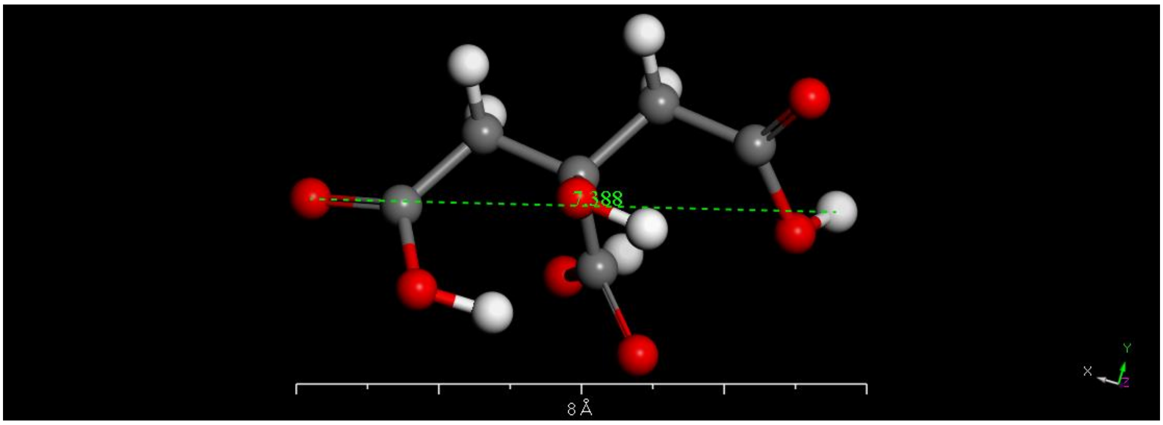

Supplementary Figure 10. Sketch of the citrate molecule. The longest distance (0.74 nm) is marked by the dashed green line.

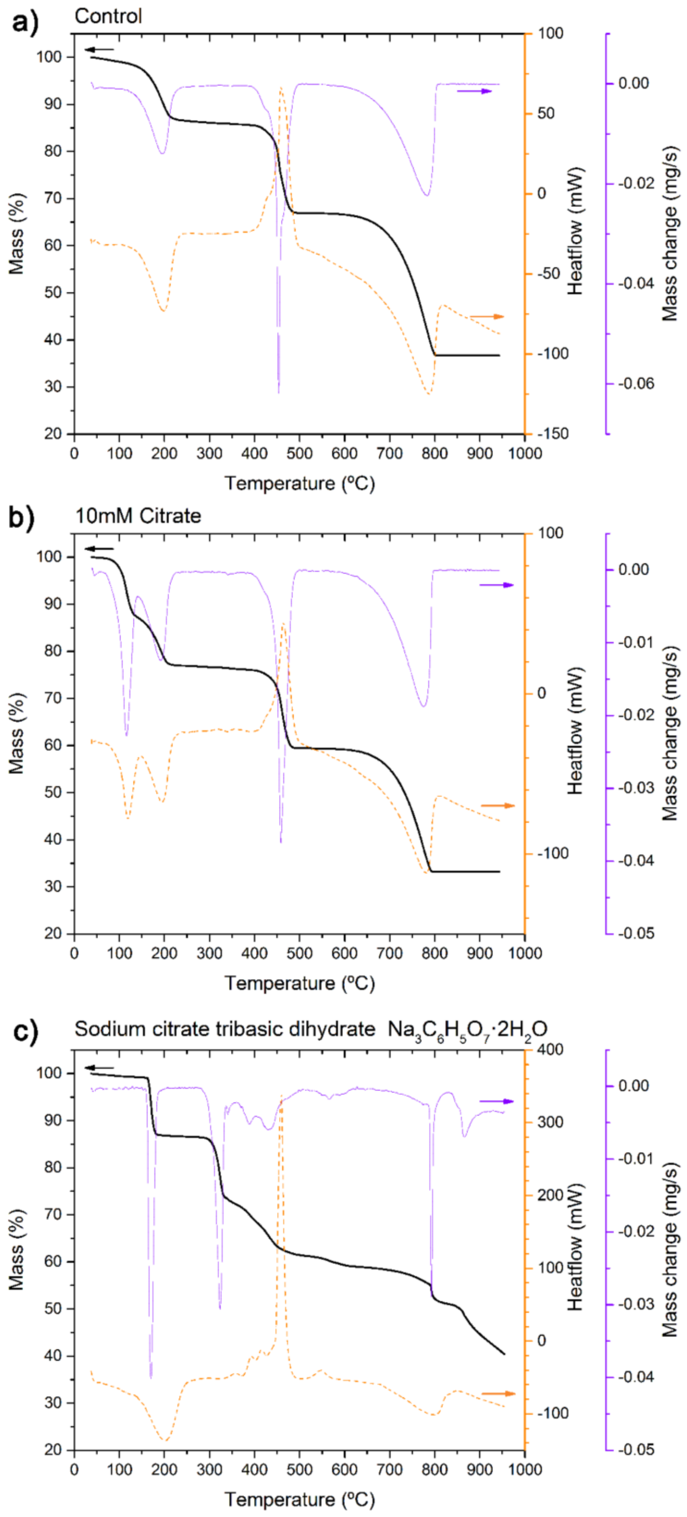

Supplementary Figure 11. Thermogravimetric analysis of calcium oxalate precipitates. TG traces of (a, b) calcium oxalate crystals precipitated (a) in the absence and (b) in the presence of 10 mM citrate. The different steps in the thermal decomposition correspond to: (1) the first weight loss at temperatures ranging 100 - 200 °C corresponds to the loss of water of

crystallization, (2) the second weight loss at temperatures ranging 400 – 530 °C, corresponds to the decomposition of anhydrous calcium oxalate, with the loss of carbon monoxide, (3) the third and final weight loss, at temperatures ranging 600 – 810 °C, corresponds to the decomposition of calcium carbonate to calcium oxide with the loss of carbon dioxide. (c) TG trace of sodium citrate, showing partial overlapping with some of the decomposition steps of calcium oxalate precipitates.
